# Using Epidemiology and Genomics to Understand Osteosarcoma Etiology

**DOI:** 10.1155/2011/548151

**Published:** 2011-03-08

**Authors:** Sharon A. Savage, Lisa Mirabello

**Affiliations:** Clinical Genetics Branch, Division of Cancer Epidemiology and Genetics, National Cancer Institute, National Institutes of Health, 6120 Executive Boulevard, EPS/7018, Rockville, MD 20892, USA

## Abstract

Osteosarcoma is a primary bone malignancy that typically occurs during adolescence but also has a second incidence peak in the elderly. It occurs most commonly in the long bones, although there is variability in location between age groups. The etiology of osteosarcoma is not well understood; it occurs at increased rates in individuals with Paget disease of bone, after therapeutic radiation, and in certain cancer predisposition syndromes. It also occurs more commonly in taller individuals, but a strong environmental component to osteosarcoma risk has not been identified. Several studies suggest that osteosarcoma may be associated with single nucleotide polymorphisms in genes important in growth and tumor suppression but the studies are limited by sample size. Herein, we review the epidemiology of osteosarcoma as well as its known and suspected risk factors in an effort to gain insight into its etiology.

## 1. Introduction

**Figure 1:**
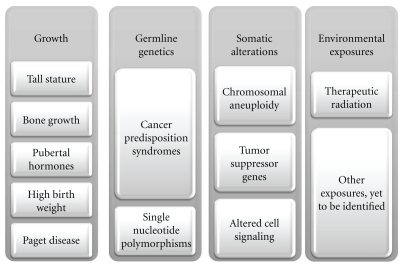
Potential contributing factors in the etiology of osteosarcoma.

Osteosarcoma, the most common primary bone malignancy, typically during the adolescent growth spurt but there is a second, smaller peak in the elderly [1]. There are a limited number of proven risk factors associated with osteosarcoma. It occurs more frequently after therapeutic radiation for a different cancer, in individuals with certain cancer predisposition syndromes, and in those with Paget disease of the bone. However, the majority of osteosarcoma cases occur in the absence of these risk factors. Numerous studies of growth and other genetic risk factors have been conducted but strong data on risk for apparently sporadic osteosarcoma are limited. The primary goal of this paper is to examine the recent studies seeking to understand osteosarcoma etiology through epidemiology and studies of germline genetics (Figure 1).

## 2. Osteosarcoma Epidemiology

### 2.1. Incidence

**Figure 2:**
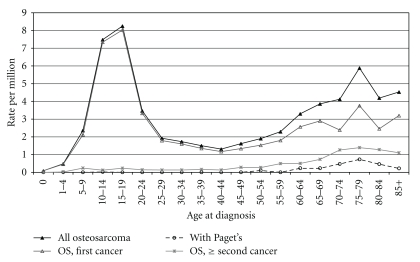
*Incidence of osteosarcoma per million population.* Data were derived from the Surveillance, Epidemiology, and End Results (SEER) program on the US population. Previously published by Mirabello et al. [2].

Osteosarcoma represents approximately 55% of child and adolescent malignant bone tumors in the US [1]. It is rarely diagnosed before the age of five, but the incidence increases with age until around puberty [1, 3–7]. This primary peak is followed by a decrease and plateau in incidence in individuals between 25 and 60 years of age (Figure 2). A second, smaller peak is observed during the seventh and eighth decades of life; this bimodal age incidence distribution of osteosarcoma is observed worldwide [8]. This is also noted in childhood and adolescent osteosarcoma where rates are relatively consistent around the world, ranging between 3 to 4.5 cases/million population/year [6, 8–13]. The rates in older persons have been less studied; current estimates are 1 to 2 cases/million population/year for persons aged 25 to 59 years and 1.5 to 4.5 cases/million population/year for persons over the age of 60 [1, 8]. Elderly individuals have a higher incidence of osteosarcoma related to Paget disease of the bone or as a consequence of treatment for a different cancer [1, 6, 14–16]. In the US and Europe, osteosarcoma incidence has somewhat increased over time in younger cases [1, 6, 12] and decreased in elderly individuals in the US [1].

In the US, using population data from the Surveillance, Epidemiology, and End Results (SEER) program, osteosarcoma incidence has been shown to vary by race based on the age of onset [17]: (1) in children and adolescents, the incidence is greatest in Asian/Pacific Islanders; (2) in individuals 25–59 years of age, the incidence is greatest in Blacks; (3) in individuals over the age of 60, osteosarcoma incidence is greatest in Whites [1]. A higher incidence of childhood osteosarcoma has been reported in Italy [18], Latin America [8], and in two African countries, Sudan and Uganda [13] compared to other populations around the world. Lower rates have been reported in Western Australia compared to the US [19]. Higher rates of osteosarcoma in the elderly have been noted in the UK and Australia [8].

It has been reported that, when a wide range of ages are combined, males are affected with osteosarcoma more frequently than females [1, 3, 4, 6, 8, 12, 19–21]. However, it has also been reported that females less than 15 years of age have slightly higher rates than males in the same age group [1, 5, 6, 14, 22–26]. In elderly patients, osteosarcoma is more common in Blacks [5] and in females, particularly those with a prior history of cancer [1]. In adolescence, incidence peaks at a later age and reaches higher rates in males (age 15–19, peak rate of 9–15 cases/million population) compared to females (age 10–14, peak rate of 6–10 cases/million population) [1, 6, 8], which suggests that bone growth, hormonal changes, and/or development associated with puberty may be involved in osteosarcoma etiology. This relationship between osteosarcoma, hormones, and growth may also partly explain the slightly higher overall incidence in males compared to females.

### 2.2. Tumor Location

**Figure 3:**
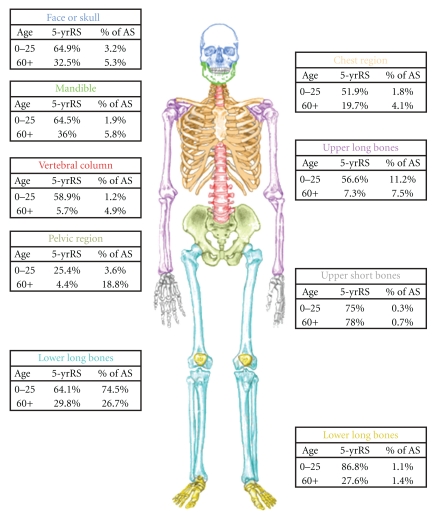
*Five-year relative survival rates (RS) by anatomic site (AS) for individuals with osteosarcoma age 0–25 years and 60+ years in the US.* The percent (%) of AS is the % of patients in that age group with osteosarcoma at that location. This figure was created using data from the SEER program in Mirabello et al. [2].

Osteosarcoma occurs most frequently in the lower long bones [1, 5, 7, 21] (Figure 3). In young patients, it most often arises at sites of rapid bone growth, the metaphyses of long bones, such as the distal femur, proximal tibia, and proximal humerus [1, 21, 27]. The occurrence of osteosarcoma most frequently in the metaphyseal area adjacent to the growth plate of long bones [28], which are the sites of particularly rapid growth during the adolescent growth spurt, reinforces the relationship between bone growth and osteosarcoma formation. There may be an increased vulnerability at these physes due to the high cell turnover during puberty. The tendency of osteosarcoma to occur in the extremity bones decreases with age, although the most common site is still the lower long bones. The lower long bones account for approximately 80% of osteosarcoma in the young patients, 27–43% in middle aged and elderly persons [1, 3, 16, 29]. In elderly patients, osteosarcomas often occur secondary to Paget's disease of the bone or some other benign bone lesion [14–16]. 

The anatomic site distributions do not vary significantly by sex or race in young patients [1] but there is more variability in middle-aged and elderly patients. This includes a higher frequency of osteosarcoma of the mandible in Blacks compared to Whites, and higher frequency of chest and upper long bone osteosarcoma but lower rates of vertebral, pelvic, or mandibular osteosarcoma in females compared to males [1].

### 2.3. Survival

Survival rates vary by age, gender, disease stage, and anatomic site (Figure 3). For children and adolescents, these rates are similar in most countries, ranging from 55–75%; although, lower rates (19–39%) have been observed in Slovakia, Estonia, and Denmark [1, 8, 30–33]. The five-year survival rate in persons aged 0–39 years was 58% in northern England [12] and 53% in Great Britain [26]. The 5-year survival in Finland for the whole study population was 58% [34]. Survival at 5 years was 57% for patients of all ages, 68% for those <41 years, and 22% for patients older than 65 years at an institute in Italy [35, 36]. Data for patients of all ages from the US National Cancer Database reported a 5-year survival of 53.9% [21], similar to the 54% reported for all age cases at the M.D. Anderson Cancer Center [37]. SEER data in the US from 1973 to 2004 showed that the relative 5-year survival rate for young-onset osteosarcoma was 61.6%; it was 58.7% for middle aged persons and 24.2% for persons over 60 years of age [1]. This paper showed a sharp decrease in survival after age 50, with rates dropping from around 50% for patients in their 50s to 17% for those in their mid-late 60s to only 11% for those in their 80s. Others have also shown that survival rates in adults over age 40 years are lower than in younger patients with rates ranging from 18–55% [16, 21, 29, 36–39].

Females have higher survival rates than males [1, 12, 21, 26, 32, 33, 40]. Disease stage is an important prognostic factor in patients with osteosarcoma at all ages, with distant disease having a much lower 5-year survival rate than localized or regional disease [1, 32]. Osteosarcoma survival rates are higher when it occurs in the short bones, and the poorest with osteosarcoma of the pelvic region and vertebral column for all ages [1, 3]. Osteosarcoma pathology has also been suggested to affect survival, though this is difficult to evaluate in most reports because many of the subtypes consist of very small sample sizes and rates are thus unstable. However, parosteal osteosarcoma has been associated with a high survival rate [21], and osteosarcoma with Paget disease a low rate [1, 21]. It has also been shown that patients with larger tumor size, metastatic disease, soft-tissue extension of the primary tumor, less tumor necrosis after neoadjuvant chemotherapy, inadequate surgical margins, or recurrence have significantly worse prognosis [7, 36–38]. 

Older patients may have unique tumor biology, for example, more axial tumors or other factors associated with a poorer prognosis, such as Paget disease (see below) and response to therapy due to age-related adjustments in therapeutic regimens, which could contribute to their worse overall survival [7, 29, 37]. Overall, osteosarcoma survival has improved over time with each decade until the 1990s, but little thereafter [1, 6, 26, 31, 32, 34, 40–43]. It has likely improved with advancements in patient care and the advent of chemotherapy, but there is still a need for novel treatment and patient management strategies shown by the lack of improvement in the last decade.

## 3. Environmental Exposures and Osteosarcoma

Studies of environmental exposures and rare cancers, such as osteosarcoma, are challenging and often limited by sample sizes. Most studies are case-control, ecologic, and/or descriptive in nature. This is because the extremely large cohorts required to study these cancers are nearly impossible to conduct. For example, even in a cohort of one million individuals, only 4 or 5 would be expected to develop osteosarcoma. Studies of environmental exposures and osteosarcoma are often combined with studies of other bone tumors, including Ewing sarcoma and others. This makes separating the potentially etiologic clues even more challenging. The reader is referred to a recent, comprehensive review of these studies [44]. Two well-studied exposures are described below.

Many years ago, it was hypothesized that fluoride could contribute to osteosarcoma risk. This was based, in part, on the fact that it is taken up by and stored in bones and on *in vitro* data which suggested that fluoride could act as a mitogen on osteoblasts [45]. Studies of fluoride exposure and osteosarcoma risk have not yielded conclusive results and have generated significant controversy. The initial ecologic studies suggested that fluoride could contribute to bone cancer etiology, but subsequent studies did not confirm this finding (reviewed by Eyre et al., 2009 [44]). A more recent study did suggest an association between fluoride and osteosarcoma in males but not in females [46] but caution was suggested in its interpretation [47].

Therapeutic radiation is a proven risk factor for osteosarcoma. It was noted to occur more frequently than expected in survivors of Hodgkin disease who received therapeutic radiation [48, 49]. Increased incidence of osteosarcoma was also noted in individuals who received Radium for ankylosing spondylitis (reviewed in [48]). However, very low doses of radiation received for medical evaluations, such as X-rays or CT scans are not associated with osteosarcoma risk (reviewed in [50]).

## 4. Growth and Osteosarcoma

Since osteosarcoma occurs most commonly during puberty, a time of rapid bone growth and remodeling, it is highly plausible that factors related to growth and development play a role in osteosarcoma etiology. Case reports of osteosarcoma occurring in individuals with acromegaly, a growth disorder caused by over production of growth hormone, lent further support to this hypothesis [51]. Early studies suggested that individuals who were longer at birth and/or taller than their peers were at increased risk of osteosarcoma [52–55]. These associations are further supported by the strong positive association of sporadic osteosarcoma and height in canines [56].

Osteosarcoma incidence is highest during puberty when endogenous sex hormones, growth hormones, and insulin-like growth factor 1(IGF1) levels are at their highest, so this biological pathway is likely to play an important role in osteosarcoma etiology. Insulin-like growth factors (IGFs) play critical roles in carcinogenesis and circulating levels are associated with risk of several cancers [57], including prostate, breast, colorectal, and lung cancer [58]. IGF1 is a potent mitogen for human osteosarcoma cell lines [59, 60]. The overexpression of insulin-like growth factor 2 (*IGF2)* and loss of IGF2 imprinting occurs in diverse cancers [61], further suggesting a role for this pathway in carcinogenesis. In addition, one small study identified single nucleotide polymorphisms (SNPs) in *IGF2R* as potential risk factors for osteosarcoma (see below) [62].

### 4.1. Height

The association between taller stature and increased risk of developing osteosarcoma was first reported in 1967 [63]. That study compared the height of 85 individuals with osteosarcoma to 202 controls between 1945 and 1965 and found that the cases were taller than controls. Two subsequent studies of 54 and 18 cases each which used percentiles of height also noted that the osteosarcoma cases were taller than expected [54, 64]. Five additional studies [53, 55, 65–67] confirmed the association of increased height and osteosarcoma risk but eight others [52, 68–74] did not find an association between height and osteosarcoma risk. The discrepancies among these studies could be a result of limited sample sizes, variable methods and control selection procedures, and thus limited statistical power. However, the largest study, a cohort study of 962 patients with osteosarcoma which used standard deviation scores to evaluate the relative height of patients, found that patients with osteosarcoma were taller than average but the association was primarily in those less than 18 years of age [53].

A recent meta-analysis of height and osteosarcoma compiled individual osteosarcoma patient data on 1067 osteosarcoma cases derived from 5 published [52, 54, 66, 67, 73] and 2 unpublished studies of height (Mirabello et al., Under Review). Cases were compared to age- and gender-matched 1000 simulated controls per case based on the 2000 US National Center for Health Statistics Growth Charts. That study showed that “taller-than-average” (51st–89th percentile) and “very tall” individuals (≥90th percentile) had an increased risk of osteosarcoma (odds ratio 1.40, 95% CI 1.13–1.73, and odds ratio 2.63, 95% CI 1.98–3.49, resp., *P*_trend_ < 0.0001).

The meta-analysis (Mirabello et al., Under Review), and a separate study of 962 patients with osteosarcoma [53], which was not included in the meta-analysis, confirm that taller stature is associated with osteosarcoma. However, the specific basis for this association is not known. For example, there are currently no data in the literature on osteosarcoma and patient height that also consider parental height. The incidence of osteosarcoma does not vary widely around the world but the average adult height varies based on country of origin [75]. Individuals with a more rapid growth velocity during puberty could potentially have increased risk of osteosarcoma because cell division is occurring more rapidly. Attaining a greater height than expected based on parental heights could also be a risk factor because of the increased bone growth required. Future studies of parental height and growth velocity will be helpful in understanding these differences.

### 4.2. Birth Weight

Numerous epidemiologic studies have evaluated associations between high birth weight and cancer. This is based on the hypothesis that high birth weight may be the result of multiple factors that are also associated with cancer. For example, IGFs are important in fetal development [76] and are also associated with cancer risk [57]. Higher birth weight has been associated with several childhood cancers, including acute lymphoblastic leukemia (ALL) [77–79], primary brain tumors [80], rhabdomyosarcoma [81], and Wilms' tumor [82, 83]. Interestingly, recent studies of ALL and Wilms' tumor suggest that the strongest associations are in females with high birth weight [82, 84]. There are also several studies suggesting associations between high birth weight and adult-onset cancers, including prostate and breast cancer [85, 86].

Review of the literature identified five published studies that evaluated the potential association between birth weight and osteosarcoma; four were null [65, 74, 79, 87], and one showed an association between higher birth weight and osteosarcoma [52]. The inconsistencies in the published data on birth weight may also be due to small sample sizes and/or inconsistent methods. A meta-analysis of the raw data from two published [52, 79] and one unpublished study of birth weight and osteosarcoma compared the birth weights of 434 individuals with osteosarcoma to age- and gender-matched controls (1000 simulated controls per case) derived from US growth charts (Mirabello et al., Under Review). In that study, individuals with high birth-weight (≥4046 g) had a marginally significant increased risk of osteosarcoma (OR 1.35, 95% CI 1.01–1.79). Females with high birth-weight, but not males, had an increased risk of OS (OR 1.49, 95% CI 1.00–2.22). Overall, the association between birth weight and osteosarcoma is not as strong as the height association, but it is similar in magnitude to other cancers. It remains conceivable that prenatal growth and factors that influence it, such as growth factors and hormones, contribute to osteosarcoma risk.

### 4.3. Paget Disease and Osteosarcoma

Paget disease of bone is a relatively common metabolic bone disorder that typically occurs in older individuals [88, 89]. It is characterized by highly exaggerated bone remodeling caused by abnormalities in osteoclast regulation. Sarcomatous transformation is rare but associated with a high mortality rate. The incidence of osteosarcoma secondary to Paget disease is not precisely known, but studies estimate that about 1% of patients with Paget disease will develop osteosarcoma [90]. In elderly persons, about half of the osteosarcomas reported are estimated to be associated with Paget disease.

The co-occurrence of osteosarcoma in the setting of abnormal bone remodeling due to Paget disease of the bone suggests that osteosarcoma may be etiologically related to abnormal bone remodeling [90, 91]. This could appear to be the case in elderly individuals but the role of abnormal bone remodeling in osteosarcoma adolescents is not known. It is conceivable that a subset of younger patients have increased genetic risk and that there could be overlap with genes that contribute to the etiology of Paget disease. Paget disease is genetically heterogeneous but recent studies implicate the RANK-NF-*κ*B signaling pathway [89]. Mutations in *SQSTM1*, a downstream scaffold protein in this pathway, are associated with familial Paget disease [92]. Many, but not all, of the associated mutations occur in the ubiquitin-associated domain of the p62 protein which is encoded by the *SQSTM1* gene [88]. Ubiquitin-associated proteins, such as p62, are important in the RANK-NF-*κ*B signaling pathway which promotes osteoclastogenesis and formation.

## 5. Genetic Risk Factors

Chromosomal aneuploidy is common in osteosarcoma cells which suggests that somatic or germline chromosomal instability could potentially predispose an individual to osteosarcoma [93, 94]. There are numerous studies of the somatic changes present in osteosarcoma cells but a common somatic defect has not yet been identified. Osteosarcoma is associated with several cancer predisposition syndromes that are caused by highly penetrant germline mutations as described in Table 1. These disorders are extremely rare and not a common cause of osteosarcoma. However, they may provide important insights into osteosarcoma etiology because the same genes that are associated with these disorders are often also disrupted in osteosarcoma tissues. Common germline genetic variants, such as SNPs, are associated with risk of numerous diseases, including cancer. The role that they play in sporadic osteosarcoma is not known, but several pilot studies have sought to understand this (Table 2).

### 5.1. Inherited, Cancer-Prone Disorders

Inherited cancer predisposition syndromes are a heterogeneous group of disorders. There are several disorders in which higher rates of osteosarcoma are noted (Table 1). Studies of these disorders have provided important clues to understanding osteosarcoma etiology.

The careful characterization of families with high rates of breast cancer, sarcomas, and other cancers by Li and Fraumeni Jr. in 1969 led to the recognition of the syndrome now known as Li-Fraumeni syndrome (LFS) [101, 102]. The classic LFS is clinically diagnosed based on family history which includes a personal history of a sarcoma diagnosed under the age of 45, a first-degree relative with cancer under age 45, and another first- or second-degree relative with cancer diagnosed under age 45 or sarcoma at any age. LFS is caused by autosomal dominant germline mutations in *TP53* [102, 103] although approximately 30% of individuals who meet clinical criteria for LFS do not have a *TP53* mutation. Additional clinical descriptions and criteria for mutation testing in individuals with suspected LFS are reviewed in [102]. The p53 protein, encoded by *TP53*, is crucial for normal cell growth, apoptosis, DNA repair, and numerous other cellular processes. The p53 gene is mutated in a majority of somatic tumor tissues, many of which disrupt the DNA-binding domain and result in a loss of tumor suppressor function [104]. Many, but not all, osteosarcomas have *TP53 *mutations but these have not consistently been correlated with disease stage or prognosis [105]. 

Retinoblastoma is a malignant retinal tumor that typically occurs prior to the age of 5. It is caused by mutations in the *RB1 *tumor suppressor gene [106]. The *RB1* gene encodes the Rb protein which is critical in normal cell cycle and differentiation processes. Loss of normal Rb function is noted in several sporadic human tumors, including apparently sporadic osteosarcoma. In addition, osteosarcoma is the most common second tumor in patients with retinoblastoma. It occurs more frequently than expected in individuals with *RB1* mutations whether or not they had radiation therapy [107, 108]. The standardized incidence ratio (SIR) for osteosarcoma occurring after retinoblastoma was 406-fold over expected for individuals who had radiation and 69-fold over expected for those who had not received radiation therapy. This suggests that both primary genetic and gene/environment interactions contribute to osteosarcoma development in the setting of a germline *RB1* mutation, and this may also be the case in apparently sporadic osteosarcoma. 

Increased rates of osteosarcoma are also present in individuals with germline mutations in DNA helicase genes, including Rothmund Thomas syndrome (RTS), Werner syndrome, and Bloom syndrome. RTS is a rare, autosomal recessive disorder caused by mutations in the DNA helicase *RECQL4* (reviewed in [109, 110]). It has a characteristic sun-sensitive rash which presents in infancy and then enters a chronic phase with poikiloderma through adulthood. Individuals with RTS may also have small stature, skeletal dysplasias, sparse hair, or cataracts. Osteosarcoma is the most common cancer in RTS; one study of 41 patients found that 32% had osteosarcoma [111]. The role of *RECQL4* in sporadic osteosarcoma is not well understood. Since the DNA helicases are critical for normal DNA structure and function, it is feasible that proteins in this family are likely to be important in carcinogenic processes and could contribute to the DNA damage and chromosomal aberrations seen in osteosarcoma cells.

Bloom syndrome, caused by autosomal recessive inheritance of mutations in the *BLM* helicase, also has a characteristic rash, but not true poikiloderma [112]. Individuals have severe pre- and postnatal growth retardation, learning disabilities, and high rates of cancers. The most common cancers are epithelial, hematopoietic, lymphoid, connective tissue, germ cell, nervous system, and kidney cancers. Three out of 168 individuals with Bloom syndrome listed in the Bloom syndrome registry were reported to have a sarcoma between 1954 and 2000 [112]. While osteosarcoma is still rare in Bloom syndrome, it is more common in this disorder than in the general population. The role of *BLM* mutations in osteosarcoma somatic cells is not well described.

Werner syndrome is a premature aging syndrome which typically presents after the first decade of life [113, 114]. It is caused by mutations in the *WRN* DNA helicase and inherited in an autosomal recessive manner. Individuals with Werner syndrome typically have characteristic “bird” facies, short stature, parental consanguinity, cataracts, atrophic skin, and signs of premature aging such as atherosclerosis. They are at increased risk of osteosarcoma as well as other malignancies [115, 116]. 

Diamond Blackfan anemia (DBA) is another inherited disorder associated with increased risk of osteosarcoma [117]. DBA is an inherited red blood cell aplasia with a broad phenotypic spectrum. Patients have variable degrees of anemia, normal leukocytes and platelets, occasional physical malformations, and increased risk of acute myelogenous leukemia, myelodysplastic syndrome, and solid tumors. Approximately 40% of patients have an identifiable mutation in a gene important in ribosomal function (*RPS19, RPL5, RPL11, RPL35A, RPS24, RPS17, *or *RPS7*). Osteosarcoma was noted in three of the 354 patients in the DBA registry in 2001 [118]. The role of these ribosomal proteins in osteosarcoma biology is unexplored. However, the higher than expected occurrence of osteosarcoma in patients with DBA is notable and warrants further study of ribosomal function in osteosarcoma. 

### 5.2. Inherited, Cancer-Prone Disorders

The inherited disorders caused by rare, highly-penetrant mutations and associated with osteosarcoma described above explain only a very small percentage of all osteosarcoma cases. It occurs more often in individuals without a family history of cancer or other medical problems. Several studies have been conducted in an effort to understand the contribution of common genetic variants, such as SNPs, to osteosarcoma risk (Table 2) although the vast majority await replication. SNPs are the most common form of genetic variation in the genome; approximately 10 million with minor allele frequencies of at least 1% are thought to be present in the genome. Most SNPs do not alter gene expression or protein function, but a subset can have subtle, yet important, biological effects. For example, an SNP in the promoter of the *MDM2* gene increases the affinity of the Sp1transcription factor which results in higher MDM2 levels and p53 pathway attenuation [119]. 

Most of the studies of SNPs and osteosarcoma conducted to date have been limited by sample size and therefore should be considered exploratory in nature (Table 2). These studies were based on *a priori* hypotheses that the genes of interest were potentially important in osteosarcoma biology. The first such study evaluated three SNPs in the promoter of the Tumor Necrosis Factor-*α* (*TNF*) gene in 63 osteosarcoma cases and 111 controls from Spain [120]. The TNF protein is a proinflammatory cytokine that has important roles in cellular proliferation and differentiation. It is also involved in bone remodeling and is a component of the RNKL pathway described above. SNPs in the TNF promoter have also been noted to affect protein expression. That study suggested that the *TNF*-238G>A was inversely associated with osteosarcoma. The *TNF*-308G>A variant was not associated with osteosarcoma. The authors also evaluated these genotypes in 47 individuals with Ewing sarcoma but did not find an association. 

In a second study, the same group hypothesized that variants in the estrogen receptor (*ESR1*), vitamin D receptor (*VDR*), and/or collagen 1*α*1 (*COL1A1*) gene could be osteosarcoma risk factors. Variants in *ESR1* could be important in osteosarcoma since estrogen is critical during puberty which is the key time of risk for osteosarcoma. The *VDR* and *COL1A1* genes are required for proper bone formation and thus, if aberrations are present, could be associated with osteosarcoma. A total of 72 osteosarcoma cases and 143 controls were evaluated. Ruza et al. found that the F*f* genotype of *VDR* was associated with increased risk of osteosarcoma (odds ratio [OR] 1.78, 95% confidence interval [CI] 1.0–3.16, *P* = 0.048) [66]. Variants in *ESR1* or *COL1A1* were not associated with osteosarcoma.

Since mutations in *TP53* cause LFS and osteosarcoma is a defining tumor of the syndrome, SNPs in *TP53* were evaluated as potential osteosarcoma risk factors in the Bone Disease and Injury Study of Osteosarcoma (BDISO), a hospital-based study of 104 cases and 74 controls [96]. Subjects genotyped were whites from the US Twelve tag-SNPs were genotyped and several inheritance models evaluated. The recessive inheritance model suggested that rs1642785 (IVS+38C>G) and rs1042522 (Ex4+119C>G, Pro72Arg) were associated with osteosarcoma risk. However, these genotypes were quite rare and this study, like those described above, was limited by its small sample size.

In a different study of the p53 pathway, Toffoli et al. genotyped the Pro72Arg (rs1042522, Ex4+119C>G) SNP in *TP53* and the *MDM2* -309 promoter SNP (rs2279744, T>G) in 201 osteosarcoma cases and 250 controls from Italy [98]. The Pro72Arg SNP in *TP53* has been associated with risk of several cancers, including lung and breast cancer (reviewed in [121]). In addition, the presence of the 72Arg allele was correlated with earlier age of cancer onset in individuals with LFS [122]. The MDM2 protein is an important regulator of TP53 function, and the -309 T>G SNP is associated with altered *MDM2* expression. LFS patients with the G allele have an earlier age of onset of cancer [122]. In addition, this *MDM2* SNP is also associated with risk of several cancers [121]. This osteosarcoma study noted that the *MDM2*-309 SNP was only associated with high-grade osteosarcoma in females. The *TP53* Pro72Arg SNP was not associated with osteosarcoma risk but an association with survival was suggested. This study did not report results of a recessive genetic model so direct comparison with the BDISO *TP53* findings in osteosarcoma was not possible.

The first study to evaluate SNPs in growth-related genes did so based on the hypothesis that since osteosarcoma most commonly occurs during a period of active growth, that variants in genes that regulate pubertal growth could be important osteosarcoma risk factors. Common SNPs in 13 growth-related genes were also evaluated as candidate risk modifiers in the BDISO. Of the 52 SNPs evaluated, two correlated SNPs in insulin-like growth factor receptor 2 (*IGF2R*, rs998075 and rs998074) were associated with increased risk of osteosarcoma (OR 2.04, 95% CI 1.29–3.24) [62]. One of those SNPs, rs998075 (Ex16+88G>A), resulted in loss of methylation in a CpG island but the impact of this alteration on *IGF2R* protein function is not known. As noted above, the IGFs are potential regulators of carcinogenesis in several cancer types and IGF1 levels have been associated with cancer risk. Followup of these findings in osteosarcoma is needed to better understand how genetic variation in *IGF2R* contributes to its etiology. 

SNPs in the 8q24 chromosomal region are being intensely studied because genome-wide association studies (GWAS) have consistently found them to be associated with risk of adult onset cancers, including prostate, breast, colon, and others [123–125]. Therefore, we recently evaluated 214 SNPs in 8q24 with a focus on the 9 SNPs which were previously associated with cancer in GWAS [100]. Ninety-nine cases and 65 controls plus an additional 1365 controls from the Prostate, Lung, Colorectal, Ovarian (PLCO) cancer screening trial were genotyped. All subjects were self-identified whites. Associations with the 9 SNPs previously associated with cancer were not noted in this study. Overall, seven SNPs were associated with osteosarcoma; the strongest result was noted for SNP, rs896324 (OR 1.75, 95% CI 1.13–2.69). These SNPs are in slightly different locations than the SNPs associated with other cancers. The details of 8q24 are still being explored, but a long-range regulator of the MYC proto-oncogene may be present in this region [126]. MYC inhibition was suggested to cause differentiation of osteosarcoma cells into mature osteocytes in a mouse model [127]. The combination of these findings suggests that further study of the 8q24 locus may yield important insights into the regulation of MYC and its role in osteosarcoma pathogenesis.

The Fas protein (gene name *FAS*, or *TNFRSF6*) is a member of the TNF receptor superfamily and plays a central role in programmed cell death. Genetic variants in FAS have been associated with increased risk of several cancers, such as melanoma, gastric, and renal cell cancer [128, 129]. Based on this, Koshkina et al. hypothesized that SNPs in *FAS* may be osteosarcoma risk factors. They evaluated four SNPs in *FAS* in 123 osteosarcoma cases and 510 controls from the US [97]. An important limitation of this study is the fact that the study subjects were of variable ethnicity; 51.2% of cases (63) and 78% of controls (398) were described as non-Hispanic whites. An SNP in exon 3 (18272A>G, dbSNP number not given) was associated with increased risk of osteosarcoma in non-Hispanic whites (OR 2.3, 95% CI 1.2–4.6). 

TGF-*β* signaling is important in the regulation of cellular proliferation. A functional polymorphisms, referred to as TGFBR1*6A, is caused by the deletion of 3 GCG triplets which code for alanine in exon 1. It is a hypomorphic variant that results in reduced TGF-*β* growth inhibitory signaling. The TGFBR1*6A variant has been associated with breast and ovarian cancer, but not consistently associated with other cancer types [130]. Thus, the potential role of this variant was explored in a study of 168 osteosarcoma patients and 168 controls [99]. The authors found that both homozygosity and heterozygosity for the TGFBR1*6A variant resulted in increased risk of osteosarcoma in the Chinese population, in a gene-dose response pattern (OR 4.6, 95% CI 2.2–7.97 and OR 2.9, 95% CI 1.59–5.34, resp.).

As a whole, the studies conducted, to date, of common genetic variants and osteosarcoma risk have yielded promising results. Their strength lies in the fact that they have evaluated genes which have a high biologic likelihood of being related to osteosarcoma etiology based on laboratory and/or other epidemiologic studies. However, the results of all the studies described above and in Table 2 should be interpreted with caution because they all have small sample sizes and limited statistical power. Future, large, multi-institutional, collaborative studies are required to obtain the necessary sample size and adequate statistical power to follow up these findings. 

## 6. Summary and Future Directions

Some progress has been made in understanding the cause of osteosarcoma, but we still have much to learn. The biggest clue generated in the study of osteosarcoma epidemiology is its association with either rapid or abnormal growth. Its occurrence primarily during the adolescent growth spurt and association with tall height at diagnosis show that bone growth is clearly an important factor. It is not known whether or not tall stature, in and of itself, is the key, or if it is taller stature than expected based on parental heights or due to height velocity during puberty. An ongoing Children's Oncology Group epidemiology study which will investigate parental height and growth charts of children and adolescents with osteosarcoma will help shed light on this question. Future clinical and laboratory studies should also carefully evaluate the complex hormonal changes that occur before, during, and after puberty. 

The association of osteosarcoma with the abnormal bone remodeling present in Paget disease also warrants more careful examination. The role of variants in genes of the RANKL-NF-*κ*B signaling pathway, which are strongly associated with Paget disease, have not been thoroughly studied as potential osteosarcoma risk factors. The case reports of the occurrence of osteosarcoma in the setting of acromegaly, a state of abnormal growth hormone production, also warrant followup. Is the literature biased by these case reports, or is there an increased risk of osteosarcoma amongst individuals with acromegaly? 

The studies of rare, but highly penetrant, cancer predisposition syndromes can shed some light on the biological mechanisms of osteosarcoma. In general, the cancers that occur in individuals with the cancer predisposition syndromes described above occur at much younger ages than in the same cancer types in the general population. The fact that several of these syndromes include osteosarcoma in the phenotype suggests that there may be common genetic mechanisms which also contribute to the apparently sporadic occurrence of osteosarcoma. It is also likely that the genetic contribution to cancers which occur in the first two decades of life, such as osteosarcoma, is greater than in cancers which do not occur until many decades later. In childhood cancer, there has been considerably less time for exposure to known and unknown environmental carcinogens. 

The contribution of environmental exposures to osteosarcoma and to other cancers of children and young adults is not known. The heterogeneity and relative rarity of these cancers create significant complexity in study design and interpretation. In addition, it is likely that a combination of environmental exposure and genetic risk factors contribute to cancer risk. Large, longitudinal, cohort studies of the cancers of children and young adults are required to address these study design issues and likely contribution of multiple factors. The International Childhood Cancer Cohort Consortium (I4C) is a multi-institutional, international collaborative group of childhood cohort studies that is working to better understand the etiology of childhood cancer [131]. However, even this large-scale effort will not be able to address osteosarcoma risk factors in detail, because of its rarity.

Like many cancers, the etiology of most osteosarcoma remains unknown. Epidemiology studies have provided many important clues, such as associations with puberty, height, and disorders of bone growth and remodeling. The genetic clues derived from the occurrence of osteosarcoma in the setting of germline mutations in genes such as *TP53* and *RB1* suggest that the genetic contribution to what appears to be sporadic osteosarcoma may also be important. Understanding potential environmental contributions to osteosarcoma risk is very challenging because of its rarity and the fact that a single environmental exposure is not likely to be the primary cause. Numerous studies are underway which seek to improve our understanding of osteosarcoma etiology and through this understanding we will be better equipped to counsel patients and refine treatment strategies.

## Figures and Tables

**Table 1:** Inherited disorders associated with increased rates of osteosarcoma.

Disorder	Gene	Chromosome	Autosomal inheritance pattern
Li-Fraumeni Syndrome	*TP53, *tumor protein p53	17p13.1	Dominant

Retinoblastoma	*RB1, *retinoblastoma 1	13q14.2	Dominant

Rothmund Thomson Syndrome	*REQL4, *RecQ protein-like 4, DNA helicase	8q24.3	Recessive

Werner Syndrome	*WRN, *Werner syndrome, RecQ helicase-like	8p12	Recessive

Bloom Syndrome	*BLM, *Bloom syndrome, RecQ helicase-like	15q26.1	Recessive

Diamond Blackfan Anemia	Ribosomal protein genes, including *RPS19, RPL5, RPL11, RPL35A, RPS24, RPS17, *and *RPS7 *	multiple	Dominant

**Table 2:** Association studies of single nucleotide polymorphisms and osteosarcoma risk. Abbreviations: SNP: single nucleotide polymorphism; OR: odds ratio; CI: confidence interval.

First Author, Year, Reference	No. cases/no. controls	Study Design	Gene	Main Finding(s)
Patiño-Garcia, 2000, [95]	63/111	Case-Control	Tumor Necrosis Factor-*α* (*TNF*)	Evaluated 3 SNPs in the promoter. *TNF-*α** -238G>A was inversely associated with risk (OR 0.17, 95% CI 0.04–0.76, *P* = 0.0095)

Ruza, 2003, [66]	72/143	Case-Control	Vitamin D Receptor (*VDR) *	3 SNPs (FokI, ApaI, TaqI) studied. FokI *Ff* genotype associated with increased risk (OR 1.78, 95% CI 1.0–3.16, *P* = 0.048)
			Estrogen Receptor (*ESR1*)	2 variants (Pvu II and XbaI) evaluated were not associated with osteosarcoma
			Collagen 1*α*1 (*COL1A1*)	1 variant studied (Msc 1) was not associated with osteosarcoma.

Savage, 2007, [96]	104/74	Hospital-based Case-Control	Tumor Protein p53 (*TP53*)	12 tag-SNPs in *TP53 *genotyped. Recessive model noted potential increased risk with rs1642785 (IVS+38C>G; OR 6.7, 95% CI 1.06-41.6, *P* = 0.04) and rs1042522 (Ex4+119C>G, P72R; OR 7.5, 95% CI 1.2–46.3, *P* = 0.03).

Savage, 2007, [62]	104/74	Hospital-based Case-Control	Insulin-like Growth Factor 2 Receptor (*IGF2R*)	Evaluated 52 SNPs in 13 growth-related genes. Two linked *IGF2R *SNPs, rs998075 (Ex16+88G>A) and rs998074 (IVS16+15C>T), associated with increased risk (haplotype OR 2.04, 95% CI 1.29–3.24, *P* = 0.006).

Koshkina, 2007, [97]	123/510	Case-Control	Fas (TNF receptor superfamily, member 6; *FAS*)	4 SNPs in *Fas* studied. Increased risk with exon 3, 18272A>G, most pronounced in non-Hispanic whites (OR 2.3, 95% CI 1.2–4.6, *P* = 0.014)

Toffoli, 2009, [98]	201/250	Case-Control	Mdm2 p53 binding protein homolog (*MDM2*)	1 SNP in *MDM2* studied, rs2279744 (SNP309T>G), was associated with high-grade osteosarcoma in females
			Tumor Protein p53 (*TP53*)	1 SNP evaluated, rs1042522 (Ex4+119C>G, P72R), was associated with survival.

Hu, 2010, [99]	168/168	Case-Control	Transforming growth factor beta receptor 1 (*TGFBR1*)	1 variant evaluated (TGFBR1*6A) was associated with increased susceptibility (OR 4.6, 95% CI 2.3–7.9, *P* = 0.002)

Mirabello, 2010, [100]	99/1430	Hospital-based Case-Control	8q24 region	Evaluated 214 SNPs, including 9 previously associated with cancer. Strongest association noted at rs896324 in additive model (OR 1.75, 95% CI 1.13–2.69, *P* = 0.01)
